# An infant case of pseudohypoaldosteronism type1A caused by a novel *NR3C2* variant

**DOI:** 10.1038/s41439-021-00173-7

**Published:** 2021-11-18

**Authors:** Saki Noda, Kohei Aoyama, Yuto Kondo, Jun Okamura, Atsushi Suzuki, Naoya Yamaguchi, Aya Yoshida, Yoshishige Miyake

**Affiliations:** 1 Department of Pediatrics, Ichinomiya Municipal Hospital, Ichinomiya, Japan; 2 Department of Pediatrics and Neonatology, Nagoya City University Graduate School of Medical Sciences, Nagoya, Japan

**Keywords:** Endocrine system and metabolic diseases, Genetic counselling

## Abstract

Pseudohypoaldosteronism type1A (PHA1A) is the renal form of pseudohypoaldosteronism with autosomal dominant inheritance. PHA1A is caused by haploinsufficiency of the mineralocorticoid receptor, which is encoded by *NR3C2*. We encountered an infant who was diagnosed with PHA1A due to hyponatremia, hyperkalemia, and poor weight gain in the neonatal period. She carried a novel heterozygous mutation (NM_000901.5: c.1757 + 1 G > C) in the splice donor site of IVS-2 in *NR3C2*.

Pseudohypoaldosteronism type 1 (PHA1), first described in 1958, is a rare disease characterized by hyponatremia, hyperkalemia, and metabolic acidosis, despite elevated aldosterone levels^1^. There are two existing forms of PHA1^2^. PHA1B (MIM #264350) is the systemic form caused by abnormalities in the epithelial sodium channel (ENaC) and is inherited in an autosomal recessive pattern. PHA1B manifests as severe life-long salt wasting resulting from resistance to aldosterone in multiple target tissues, such as the sweat glands, salivary glands, colonic epithelium, and lung. PHA1A (MIM #177735) is the renal form and shows autosomal dominant inheritance; it is caused by haploinsufficiency of the mineralocorticoid receptor (MR), which is encoded by *NR3C2* (nuclear receptor subfamily 3 Group C member 2, MIM600983) on chromosome 4q31. The main symptoms of PHA1A are dehydration, vomiting, and failure to thrive. In PHA1A, aldosterone resistance is confined to the kidneys, and treatment with salt supplementation is generally unnecessary by the age of 1–3 years. Herein, we report a novel variant of *NR3C2* in a Japanese infant and her father with PHA1A.

The patient was delivered by cesarean section at week 38 of gestation because of the pelvic position. Her body weight at birth was 2860 g. She was admitted to the hospital shortly after birth because of breathing problems and confirmed spontaneous pneumothorax. She was intubated from days one to six, and the infusion was terminated on day 11. She was discharged on day 17 with a body weight of 2506 g, showing an average weight gain of 31 g/day in the previous five days. At her one-month checkup on day 28, she weighed 2502 g and had not gained any weight. Laboratory examination to investigate her poor weight gain revealed hyponatremia (120 mmol/l; normal range, 138–146 mmol/l) and hyperkalemia (6.9 mmol/l; normal range, 3.6–4.9 mmol/l); the patient was readmitted to our hospital. Her arterial blood gas analysis indicated a pH of 7.313, mild acidosis, and low bicarbonate (15.7 mmol/l; normal range, 21–28 mmol/l). Results of spot urine analysis showed elevated fractional excretion of sodium (FENa) (1.78%, median [25th–75th percentiles] for the same age, 0.44 [0.30–0.57] %) but normal fractional excretion of potassium (FEK) (13.2%, median [3rd-97th percentiles] for infants, 12.2 [3.8–27.4] %)^3,4^. Normal saline and 10 mg/kg hydrocortisone were administered because of concerns about shock due to extreme lack of vigor, poor feeding, and electrolyte abnormalities. Results of endocrinological evaluation at the time of readmission confirmed extremely high levels of both plasma renin activity (319.2 ng/ml/h, mean ± standard deviation for the same age, 5.70 ± 2.97 ng/ml/h) and plasma aldosterone (30400.9 pg/ml, mean ± standard deviation for the same age, 381.6 ± 209.5 pg/ml)^5^. In addition, adrenocorticotropic hormone (ACTH) was 49.2 pg/ml (normal range, 7.2–63.3 pg/ml) and cortisol 36.3 μg/dl (normal range, 4.5–21.1 μg/dl) at that time; there was no evidence of decreased adrenal function. We diagnosed her with PHA1 and added oral salt (8.8 mEq/kg/d). After the child’s hospitalization for hyponatremia, a history of pseudohypoaldosteronism was revealed in her father, aunt, and paternal grandmother, all of whom had taken salt as infants (Fig. 1a). According to her father, he had never been diagnosed with hypertension during occupational health examinations. Although hyponatremia and hyperkalemia were observed from younger than 10 days of age at the time of her first admission (Fig. 1b), no further detailed examination was performed. Her serum sodium level returned to the normal range after starting oral salt therapy, and her body weight increased. Salt supplementation was discontinued when she was eight months old.

**Fig. 1:**
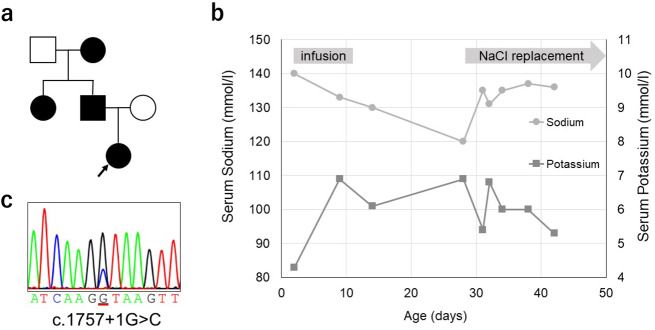
Pedigree diagram, electropherogram, and course of electrolytes. **a** Pedigree diagram. The patient’s grandmother, aunt, and father had a confirmed history of PHA1A, as represented by either dark squares (males) or circles (females). **b** Serum sodium and serum potassium levels in the neonatal period. **c** Results of Sanger sequencing for *NR3C2* in the patient.

We obtained consent for *NR3C2* gene analysis from her parents. The experimental protocols were approved by the Ethical Committee for the Study of Human Gene Analysis at Nagoya City University Graduate School of Medical Sciences (Control Number: 70-00-0200). We analyzed the *NR3C2* gene and identified a novel heterozygous mutation of the splice donor site in IVS-2 (NM_000901.5: c.1757 + 1 G > C) in the patient (Fig. 1c). Her father, who had a history of pseudohypoaldosteronism, harbored the same *NR3C2* mutation, whereas her mother did not. This variant is not present in Human Genetic Variation Database (HGVD; http://www.hgvd.genome.med.kyoto-u.ac.jp) or Genome Aggregation Database (gnomAD v2.1.1; https://gnomad.broadinstitute.org/). This mutation is located at chr4:148,435,103 in the University of California Santa Cruz genome browser for human genome assembly (http://genome-asia.ucsc.edu/human GRCh38/hg38), and its location is highly conserved among various species. The mutation is classified as pathogenic according to the American College of Medical Genetics and Genomics guidelines, as it meets the classification criteria of PVS1, PM2, and PP4 for pathogenic variants^6^. A different mutation at the same splice donor site (c.1757 + 1 G > A) has been reported in a patient with PHA1A^7^. Based on these facts, we concluded that the pseudohypoaldosteronism in the infant and her father was caused by this *NR3C2* mutation.

PHA1A tends to have milder symptoms and a later onset than PHA1B. PHA1A is commonly diagnosed at approximately 2–4 weeks of age, sometimes within 10 days of age, because of failure to thrive^2,7–9^. In our case, hyponatremia and hyperkalemia were observed younger than 10 days of age, though the data were obtained during the infusion, and hyponatremia progressed further after the infusion was stopped. *NR3C2* mutations are inherited in approximately 70% of PHA1A cases, and de novo *NR3C2* mutations are relatively rare^10,11^. As unexplained early death in PHA1A families suggests that PHA1A may be fatal^8^, it is important to obtain a family history and provide appropriate interventions, such as prophylactic salt administration, when hyponatremia is observed in the neonatal period, even in mild cases, as in the present patient. Furthermore, PHA1A cannot be ruled out, even if there is a temporary increase in weight, such as that observed during the first predischarge neonatal period. One unusual aspect of our PHA1A case was the complication of spontaneous pneumothorax, but there are no similar reports, and we considered it an incidental complication. Adults with PHA1A are considered to have lifelong elevated renin activity and high angiotensin 2 and aldosterone levels, yet they are clinically indistinguishable from unaffected individuals^8^. Biochemical data for the adults in the family with this variant could not be evaluated in this study.

In conclusion, we identified a novel *NR3C2* mutation in a PHA1A patient and reported the electrolyte course in the neonatal period.

## HGV database

The relevant data from this Data Report are hosted at the Human Genome Variation Database at 10.6084/m9.figshare.hgv.3106.
